# Systematic discovery of regulatory motifs in *Fusarium graminearum *by comparing four *Fusarium *genomes

**DOI:** 10.1186/1471-2164-11-208

**Published:** 2010-03-26

**Authors:** Lokesh Kumar, Andrew Breakspear, Corby Kistler, Li-Jun Ma, Xiaohui Xie

**Affiliations:** 1 Broad Institute, Cambridge, MA, USA; 2 USDA ARS Cereal Disease Laboratory, University of Minnesota, St Paul, MN, USA; 3 Department of Computer Science, Institute for Genomics and Bioinformatics, University of California, Irvine, CA, USA

## Abstract

**Background:**

*Fusarium graminearum (Fg)*, a major fungal pathogen of cultivated cereals, is responsible for billions of dollars in agriculture losses. There is a growing interest in understanding the transcriptional regulation of this organism, especially the regulation of genes underlying its pathogenicity. The generation of whole genome sequence assemblies for *Fg *and three closely related *Fusarium *species provides a unique opportunity for such a study.

**Results:**

Applying comparative genomics approaches, we developed a computational pipeline to systematically discover evolutionarily conserved regulatory motifs in the promoter, downstream and the intronic regions of *Fg *genes, based on the multiple alignments of sequenced *Fusarium *genomes. Using this method, we discovered 73 candidate regulatory motifs in the promoter regions. Nearly 30% of these motifs are highly enriched in promoter regions of *Fg *genes that are associated with a specific functional category. Through comparison to *Saccharomyces cerevisiae (Sc) *and *Schizosaccharomyces pombe (Sp)*, we observed conservation of transcription factors (TFs), their binding sites and the target genes regulated by these TFs related to pathways known to respond to stress conditions or phosphate metabolism. In addition, this study revealed 69 and 39 conserved motifs in the downstream regions and the intronic regions, respectively, of *Fg *genes. The top intronic motif is the splice donor site. For the downstream regions, we noticed an intriguing absence of the mammalian and *Sc *poly-adenylation signals among the list of conserved motifs.

**Conclusion:**

This study provides the first comprehensive list of candidate regulatory motifs in *Fg*, and underscores the power of comparative genomics in revealing functional elements among related genomes. The conservation of regulatory pathways among the *Fusarium *genomes and the two yeast species reveals their functional significance, and provides new insights in their evolutionary importance among Ascomycete fungi.

## Background

Collectively, fungal species within the genus *Fusarium *are among the most important group of plant pathogens, causing disease in nearly every agriculturally cultivated plant [1]. Mycotoxins produced by *Fusarium *species also pose a significant hazard to food safety and human health [2,3]. The economic and scientific importance of these fungi has led to whole genome sequencing and functional studies of multiple economically important and phylogenetically related *Fusarium *species including, *F. graminearum (Fg)*, *F. verticillioides (Fv)*, *F. oxysporum (Fo)*, and *F. solani *[4-8]. Such rich genomic resources enable the discovery of many biological features related to the genetic mechanisms of organism adaptation and pathogenicity for these species [9-11].

As functional elements, transcription factor binding sites often evolve at a much slower rate than neutral sequences, and therefore they often stand out from the surrounding sequences by virtue of their greater levels of conservation. This property enables the recognition of these conserved elements through comparative genomics. Previous work has demonstrated the power of comparative genomics for discovering novel regulatory motifs in yeasts [12], *Plasmodium *[13], flies [14], mosquitoes [15] and mammals [16-18]. Even though there is growing body of knowledge about pathogenicity related gene regulation in the *Fg *genome, including spore germination [10] and adaptation to the host environment [11], very little is known about the transcription factors involved in the process and the regulatory elements targeted by these transcription factors. Comparative *Fusarium *genomics enables the generation of large-scale unambiguous alignments [8] among the four sequenced *Fusarium *genomes. The total divergence among these four genomes, measured by the total branch length of the tree connecting the four species is 0.35 (i.e. 0.35 substitutions per base, roughly the distance between human and dog). This distance is similar to that used successfully for identifying regulatory elements in other species [12,18].

**Figure 1:**
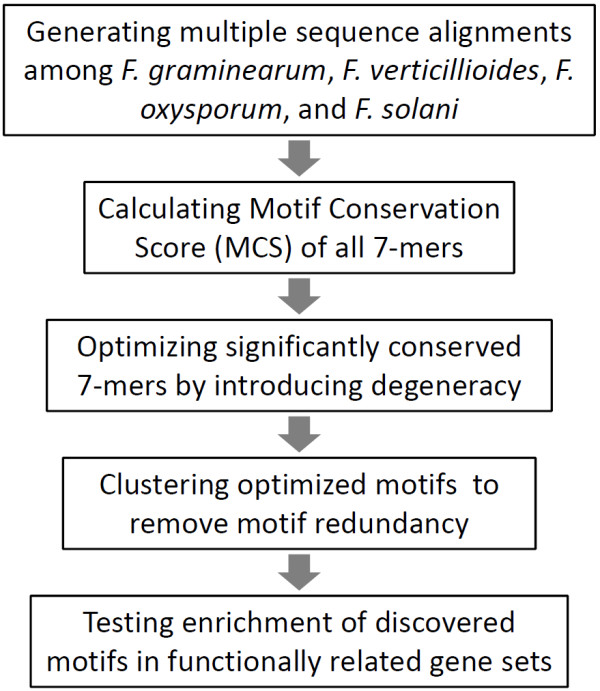
**Overview of the computational pipeline used for motif discovery**.

Here we describe a comparative genomics approach to systematically discover potential regulatory elements based on the sequence conservation of the non-coding regions among four sequenced *Fusarium *genomes (Figure 1). The results were supported by several lines of evidence, including: 1) co-regulation of subsets of genes that share the common regulatory elements in the promoter regions based on microarray expression data; 2) conservation of some of the potential transcription factor (TF) binding sites, the TFs themselves and the target genes regulated by these TFs compared to transcriptional regulatory networks described in the two model systems, Saccharomyces cerevisiae (Sc) and Schizosaccharomyces pombe (Sp); and 3) identification of a splice donor site as the top intronic motif. As the first systematic survey of potential regulatory elements in *Fusarium *species, our study revealed that the most conserved regulatory elements from both upstream and intronic regions span distantly related fungal species, indicating that these elements are under strong functional constraint by negative selection and confirming that many discovered motifs likely have an essential biological function.

## Results

### Motif discovery by comparing four *Fusarium *genomes

#### Alignment

The whole-genome alignments of the four *Fusarium *species were generated using PatternHunter (see Methods) and further divided into three functionally distinct genomic regions - promoters, downstream, and introns. The gene annotation of *Fg*, the first sequenced [5] and better-annotated [6]*Fusarium *genome, was used as a reference. Because most of the *Fg *genes are computationally predicted and thus do not have well-defined transcriptional start or stop sites, we defined the promoter region to be up to 600 bp upstream of the translation start site (ATG) and not overlapping its neighboring gene. We chose 600 bp based on the mean intergenic distance of ~1200 bp in *Fg*. Similarly, we defined the downstream region of a gene to be up to 600 bp downstream of the stop codon without overlapping any other genes or potential promoter regions. Based on this definition, the downstream regions could be enriched for 3'UTR sequences, for which little is known in *Fusarium *genomes. To avoid redundancy, we further post-processed the promoter and downstream datasets to make sure that each genomic region is represented only once. Overall, the promoter data set contains 3.4 million aligned bases, whereas the intronic and downstream sets contain 1.3 and 2.3 million aligned bases respectively. Some segments could not be aligned in all four species, primarily because they represent new sequences recently inserted into the *Fg *genome, ancestral sequences deleted in one of the other species, or missing sequences in one of the draft genomes.

#### Motif discovery

A score metric called Motif Conservation Score (MCS) [18] was used to evaluate the conservation properties of all 7-mer motifs within each of these three datasets (see Methods and Additional file 1). We focused our initial efforts on 7-mers because most regulatory motifs are in the range of 6-10 bps, with 6-mers tending to give too many random matches, whereas 8 or higher-mers too few (Note also that 7-mers are only used for initial screening; the exact lengths of the motifs are actually determined later when we introduce degeneracy to motifs). For each dataset, we exhaustively searched for the presence of 7-mers and obtained two values for each of them (i) the total occurrences in the reference genome *Fg *(*N*), and (ii) the number of occurrences that are conserved among all four genomes in the aligned regions (*k*). We called a motif occurrence conserved if the identical heptamer in the aligned regions occurs within a window of -5 bp to +5 bp compared to the reference position. The 5 bp extension at each side was introduced to account for potential errors in local sequence alignments. We next evaluated the MCS value for each heptamer, which essentially represents the difference in conservation of a heptamer from the expected conservation of a random heptamer (see Methods). For instance, the 7-mer CACGTGA occurred *N *= 264 times in the *Fg *genome of the promoter dataset. Among the 264 sites in *Fg*, k = 151 are also conserved in the corresponding orthologous regions of the three other species, corresponding to a conservation rate (C.R.) of 57%. By contrast the conservation rate for a random 7-mer in the promoter dataset is only *p*_0 _= 3%, which indicates that the CACGTGA motif shows a 19 fold enrichment in conservation rate, corresponding to an MCS = (*k-Np*_0_)/sqrt(*Np*_0_(*1-p*_0_)) = 51 *s.d*.

**Figure 2:**
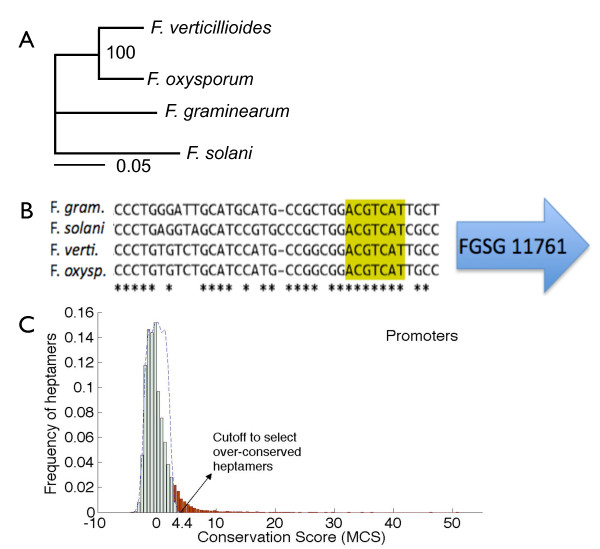
**Conservation properties in *Fusarium *promoters**. (A) Phylogenetic relationship among four *Fusarium *species (unit of branch length is substitution per site). (B) Example of aligned sequences upstream of gene FGSG_11761 in *Fg*. The starred base positions are conserved across all four species. The sequence contains the candidate motif M3: ACGTCAT, discovered through our methods. (C) Excess conservation of heptamers in the promoter region of *Fusarium *species. The Motif Conservation Score (MCS) distribution indicates a bias towards excess conservation. The dashed curve is a hypothetical MCS distribution when the right and left sides are symmetric around zero. The excess conservation, outlying this dashed curve, is highlighted in red. The MCS value at the termination point of this dashed curve is used as the cut off to select the set of over-conserved heptamers.

The distribution of the MCS values in each region is biased towards positive values (Figure 2 and Additional file 1, Figure S1), indicating that many heptamers are more conserved than would be expected from a completely random case. The lowest MCS value of a heptamer is -4.4, -4.1 and -2.4 in the promoters, downstream regions and introns respectively. We selected the absolute value of the lowest score in the promoters as the threshold to select over-conserved and thus potential regulatory motifs in all regions. If the sequences in the alignment evolved neutrally, the MCS scores would be symmetric and centered at zero. Thus, we would expect to see no heptamers with MCS values higher than the selected threshold for each region. In contrast, the selection step yielded 688 heptamers in the promoter region, 326 heptamers in the downstream region and 234 heptamers in the intronic region, corresponding to 4%, 2% and 1.4% of all heptamers in three regions respectively. We noticed that the heptamers identified from each region are largely non-overlapping with only 50 heptamers shared by all three regions (Additional file 1, Figure S2), suggesting that the identified motifs are mostly region-specific. The 50 shared heptamers likely result from partial overlap of functional regions due to incorrect annotation of a subset of genes, true signals shared by all three regions, or simply random matches.

#### Adding Degeneracy

Regulatory motifs typically contain certain variations without greatly affecting the overall binding affinity, as many of the selected heptamers are slight variations of each other. Therefore, we sought to identify the true motifs associated with the discovered heptamers by introducing degeneracy into certain positions of the heptamers. If the motif, after introducing the degeneracy, is a closer representation of a regulatory element, the MCS score of the modified motif should be higher. Therefore, we developed a greedy algorithm to systematically search for the pattern of degeneracy that can lead to an increase in the MCS score of the modified motifs (see methods and Additional file 1). Briefly, the program randomly selected a base position in the heptamer and added a degenerate character that is consistent with the nucleotide at that position. If this change increased the MCS in the updated motif, we repeated this process for one more base position. As this greedy approach is susceptible to local maxima, we repeated it ten times and selected the degenerate motif with the highest MCS from all the trials. We used this approach on the heptamers in the decreasing order of their MCS values and ignored a heptamer if it could be specified by one of the degenerate motif derived from earlier heptamers. This step finally gave 326 motifs in the promoter regions, 206 in the downstream and 114 in the intronic regions. Some of the heptamers were able to increase their score by as much as 300%. ACGCGTC in promoters, for example, increased its MCS from an original value of 14.3 to 40.6 as it degenerated into nCGCGnC ('n' represents any nucleotide). The complete list of degenerate motifs is available from our project website http://www.broadinstitute.org/annotation/genome/fusarium_group/SupplementalMaterials.html.

The MCS score measures the conservation across all sites of a motif; therefore it should not be very sensitive to the specific definition of promoters we used as long as the distribution of the motif in the promoters is not biased toward long distances. To illustrate this, we generated two additional promoter datasets using the distance of 400 bp and 1000 bp respectively for the motif discovery. For the 400 bp dataset, we discovered 288 motifs (prior to clustering), of which 286 are present in the 326 motifs discovered in the 600 bp dataset. For the 1000 bp dataset, we discovered 394 motifs, which include all of the 326 motifs discovered in the 600 bp dataset. For these 394 motifs, more than 80% of all the sites are located within 600 bp from gene starts with the highest density located at 70 bp from gene starts (Additional file 1, Figure S3). This suggests that the 600 bp is a reasonable choice for the definition of promoter regions, although we note that distant motifs preferentially located far away from gene start sites are likely to be missed with this definition.

#### Clustering

The degenerate motifs, thus derived, were still redundant. Our next approach to more accurately predict potential regulatory elements was to further cluster degenerate motifs using the Pearson correlation as a similarity measure for each pair of motifs (see Additional file 1). The Pearson correlation computes the sequence similarity between the equivalent position weight matrices [19] and quantifies it with a score from -1 to +1; with +1 for identical motifs. We grouped motifs in the same cluster if they were at least 0.75 correlated with the highest MCS motif in the cluster. From this final stage of our computational pipeline, we identified 73 clusters in promoters, 69 in the downstream and 39 clusters in the intronic region. Each cluster is represented by the highest scoring motif within that cluster. The list of candidate motifs from each cluster is shown in Table 1 and Additional file 1, Table S1.

**Table 1:** Top 30 discovered motifs in the promoter regions as ranked by MCS scores

ID	Top Motifs	Genome wide statistics for the aligned region				Functional category enrichment*			
		
		*N*	*k*	**C.R**.	MCS	Most enriched functional category	Genes in functional category	Motif genes in Fun- Group	Enrichment Score
M1	CCCCnC	4734	1462	0.30	93.5	Nucleolus 1	128	75	11.9
M2	CACGTG	623	315	0.50	66.4	Ribosome 2	88	47	24.3
M3	CGTCAY	1970	468	0.23	41.6	Ribosome 2	88	53	5.0
M4	CGCGnC	2195	461	0.21	40.6	Nucleus 2	341	173	9.6
M5	CGCCnC	3007	525	0.17	37.2	-	-	-	-
M6	CCCCnG	2985	509	0.17	36.0	-	-	-	-
M7	CCnCCA	5444	767	0.14	35.4	Nucleus 2	341	178	7.9
M8	CTCCnC	4829	673	0.13	35.2	Peroxisome 2	32	24	5.4
M9	CnCCGMC	1857	357	0.19	33.8	Nucleolus 1	128	43	12.2
M10	CnCnCCC	4201	565	0.13	31.8	Nucleus 3	159	110	6.2
M11	CCAATnA	1105	212	0.19	30.6	-	-	-	-
M12	GCnnCGC	2624	385	0.14	29.3	Cytoskeleton 1	22	15	5.3
M13	ACGYSAC	797	173	0.21	27.9	Nucleus 2	341	150	6.7
M14	CCSGCC	1520	258	0.16	26.5	-	-	-	-
M15	CnGCnCC	3170	421	0.13	26.0	-	-	-	-
M16	CnTCnCC	5870	636	0.10	26.0	-	-	-	-
M17	ACCCCG	761	152	0.19	25.9	-	-	-	-
M18	CGGnCCG	369	93	0.25	25.2	-	-	-	-
M19	AAAAA	6137	668	0.10	24.5	Nucleolus 1	128	113	19.0
M20	CCnCnTC	5505	545	0.09	24.4	-	-	-	-
M21	AAAWTTY	853	169	0.19	23.7	Nucleolus 1	128	101	31.4
M22	TGCCCC	777	139	0.17	22.9	Nucleus 2	341	98	5.4
M23	GnGGCT	2449	321	0.13	21.5	-	-	-	-
M24	GCGCnC	1673	239	0.14	21.2	Nucleus 2	341	196	6.4
M25	TCCCnC	4141	432	0.10	20.8	-	-	-	-
M26	YGATAAG	393	85	0.21	20.2	Nucleolus 1	128	38	10.9
M27	CGACnnC	3725	399	0.10	19.9	-	-	-	-
M28	CCGCnGn	1874	245	0.13	19.7	-	-	-	-
M29	CCTCGGY	381	80	0.20	18.9	Peroxisome 2	32	22	13.5
M30	AnnCCAC	3708	373	0.10	18.6	-	-	-	-

Each motif shown here is the representative motif (i.e. the one with highest MCS score) from each cluster. *N *and *k *respectively represent the number of total occurrences in *F.g*. and the number of occurrences conserved in all four Fusarium genomes. C.R. is the conservation rate of the motif (= *k*/N) and MCS is the motif conservation score. * The promoter enrichment statistics is for the motif with the highest enrichment score (10^th ^column) within each cluster shown in the 1^st ^column. The names of the functional categories are derived from the GO annotation of *F. g*. The number associated with each category represents the cluster as identified by the k-means clustering on the expression data within each category. Enrichment score is in the unit of -log_10_(p-value).

### Association with functional gene clusters

Since very little is known about the constituents of regulatory pathways in *Fusarium *species, we searched for potential associations of promoter motifs with specific gene functions using a combination of Gene Ontology (GO) annotation [20] and expression profiles [10]. For each motif, we searched for the set of genes that contain the motif or its reverse pair in its promoter region. Each gene set associated with particular promoter motif is divided into GO category group per their cellular functions. Genes within each GO category group were further sub-grouped into clusters of genes with similar expression profiles using K-means clustering of previously published expression data for conidial germination [10]. Gene expression was measured in fresh conidia (0 h) and at three other developmental milestones: spore swelling (2 h), germ tube emergence and elongation (8 h) and hyphal branching (24 h).

The GO cellular annotation provides a total 21 functional categories over 13332 *Fg *genes. Each category was further divided into 2 to 5 clusters within each GO category through K-means analysis and resulted in a total of 68 sub-clusters (Called FunGroup). A functional survey for each cluster was carried out using MIPS FunCat (5, 6). Gene lists, expression profiles and functional analysis for each cluster can be found on our project website shown above.

**Figure 3:**
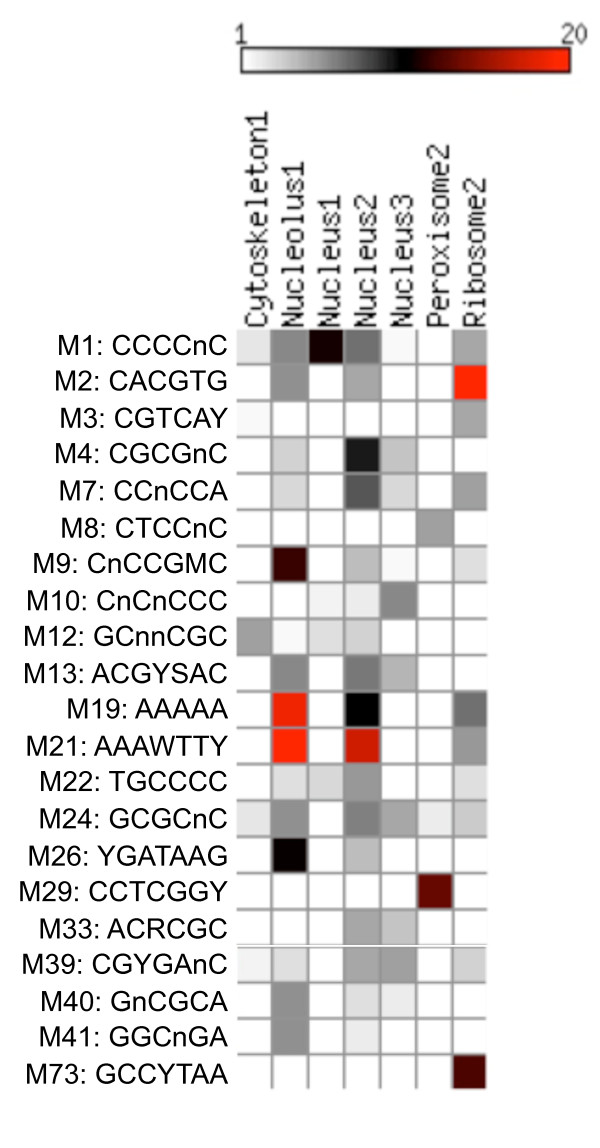
**Enrichment of promoter motifs in functional gene clusters**. Many of the discovered motifs are enriched in the upstream regions of genes grouped on the basis of GO annotation and expression profiles. This enrichment is quantified by a *P-value *derived using hyper-geometric distribution (see methods). The pseudo-colors in the cells represent -log_10 _(Hypergeometric *P-value*). The figure shows the cluster and the motif with the highest enrichment within that cluster. Only those clusters with a minimum enrichment score of 5.0 are shown. The columns represent the gene sets derived using GO annotation and clustering with the expression data within each GO category.

Even though, only limited expression data was used for this analysis, the resulting clusters reflect the functional association expected during spore germination. For example, cytoskeleton cluster 1 is enriched for genes involved in budding, cell polarity and filament formation (MIPS category 43.01.03.05; *P *= 5.85E-21), such as actin and microtubule cytoskeleton genes necessary for polarized growth in filamentous fungi [21]. As expected, genes belonging to this cluster are up-regulated between 2 h and 8 h, following the switch to polar growth and germ tube emergence. Even though the FunGroups resulting from this analysis only capture part of the transcriptome, their significant association confirmed the potential functionality of the promoter motifs discovered by our computational pipeline. The significance of enrichment for motif containing genes within each FunGroup was quantified using the hypergeometric distribution (see methods). Even though the promoter motifs are clustered based on sequence similarity, we tested each motif individually within all the 68 genes subgroups to increase specificity, knowing that a single base difference may produce slightly different target gene set. At P-value < 10^-3^, 40 motif clusters had at least one motif that is significantly enriched. At P-value < 10^-5^, 22 motif clusters (30%) showed strong enrichment (Figure 3). Notable examples include the palindrome motif M2 (CACGTG), strongly enriched in the second expression cluster of the Cellular Ribosome GO category, and the M29 motif (CCTCGGY), highly enriched in the second expression cluster of the Cellular Peroxisome category. Some motifs, like M1, M19 and M21, are enriched in multiple functional categories, suggesting their essential roles in gene regulation.

We further tested the significance of the enrichment between the promoter motifs and FunGroups through a control experiment, in which we generated a set of control motifs by randomly permuting the bases within each discovered promoter motif. The enrichment analysis on the control motifs resulted in only two enriched clusters (2%) (P-value < 10^-5^), much less than 22 as for the discovered motifs (30%). This test may suggest that the false discovery rate is below 9%, (P value < 10^-5^), and the enrichment of the motif containing genes within FunGroups are likely due to biologically bona fide associations.

### Association with known transcriptional pathways

**Figure 4:**
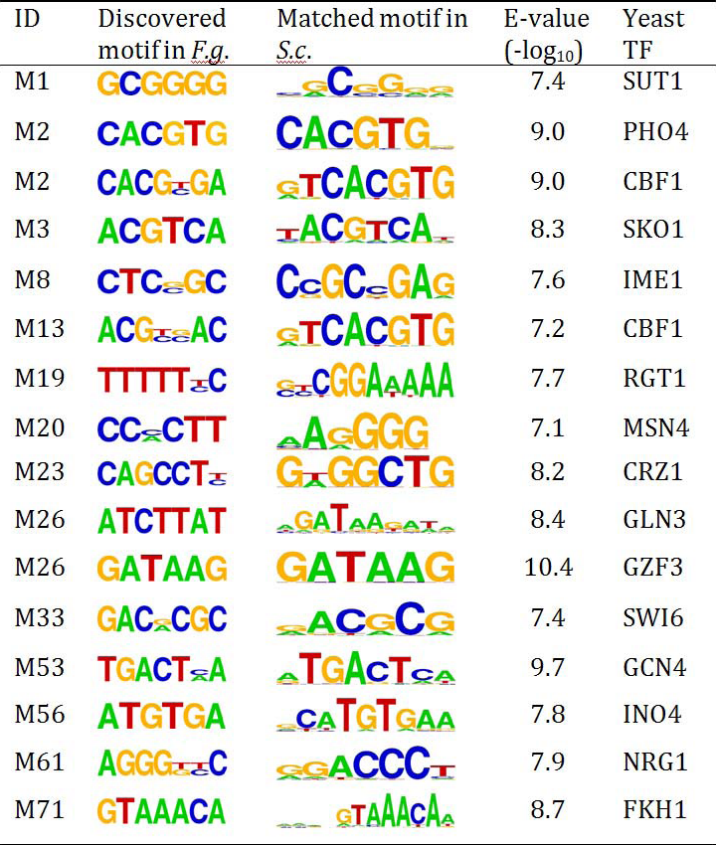
**List of discovered F.g. motifs that match known motifs in *S. cerevisae***. Motif sequence similarity was measured by Pearson correlation coefficient. E-value represents the number of hits expected to occur by chance when comparing the *F.g*. motif to all known *S.c*. motifs.

Furthermore, we are interested in understanding specific functions and potential regulatory pathways associated with the identified promoter motifs in *Fg *by comparing the motif and motif containing genes to known TF binding sites and their target genes. We have focused such comparison with two yeast systems *Sc *and *Sp*, and extended this to other known ascomycete fungal TFs. Based on the motif comparison [22] (see Additional file 1), we found 16 (21%) discovered motifs at E-value < 10^-7 ^(Figure 4) or about half of the motifs at, E-value < 10^-5 ^share sequence similarity to the binding sites reported in *S. cerevisiae *(*Sc*) [23]. If the sequence conservation truly reflects the functional association of these motifs, we were interested in determining whether these motifs are regulated by orthologous TFs and whether the target genes also are conserved in these distantly related fungal species.

#### Phosphate metabolism

**Figure 5:**
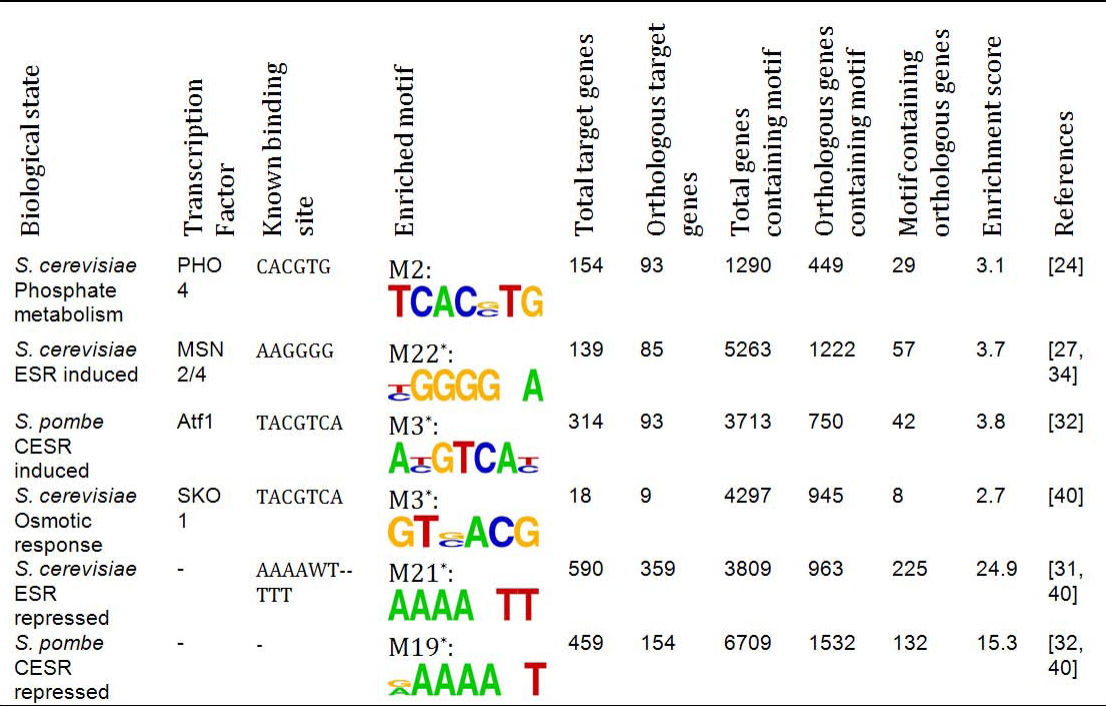
**Conservation of both binding site and target genes between Fusarium and yeast**. We found 2573 genes out of total 13332 *Fg *genes (19%) with orthologous pairs in *Sc *by BLAST program (1e-5). Similarly, we found 2378 *Fg *genes (18%) with orthologous pair in *Sp*. We checked the presence of our motifs enrichment in the functional sets of genes identified through previous studies (references in the last column) or by their sequence similarity to transcription factors (Figure 4). * indicates that the corresponding motif is also the most significantly enriched motif among all discovered degenerate motifs for the set.

The most significant match between *Fg *motifs and the *Sc *TF binding site is the motif M2 (CACGTG), which has the second highest MSC score in our list and matches the Pho4 motif in *Sc*. *Pho4 *is a basic helix-loop-helix (bHLH) TF that functions during phosphate (Pi) starvation [24]. In high phosphate growth conditions *Pho4 *is phosphorylated and present only in the cytoplasm. Under phosphate starvation, dephosphorylated *Pho4 *enters the nucleus and activates *pho *genes involved in response to phosphate starvation [25]. There is a *Pho4 *ortholog in each *Fusarium *genome (FGSG_00545, FOXG_00510, JGI_32105, and FVEG_01003). More than 60% of the Pho4 interacting genes in *Sc *[26] have a homologous gene in *Fg *(BLAST 1e-5) and the potential binding site M2 is significantly enriched among this set of genes (Figure 5). As demonstrated for both *Neurospora crassa *(*Nuc1*) [27] and *Aspergillus nidulans *(*PalcA*) [28], our study confirms that the *Pho4 *orthologs regulate the PHO system in filamentous fungi through the same TF and TF binding site, strongly suggesting the biological importance of this particular pathway.

Interestingly, through the GO category enrichment test, motif M2 (CACGTG) is highly enriched in the upstream promoter regions of genes in ribosome cluster 2 (Figure 3). Within this cluster, 44 out of 47 genes are predicted ribosomal proteins. Remarkably, we observed the expression profile for genes contained in ribosome cluster 2 peaking sharply at 2 and 8 h, periods of spore activation and germ tube emergence where a high level of ribosome biogenesis would be required to cope with an increased demand for nascent proteins. Even though, the components of the ribosome have not been identified as targets of *Pho4 *in yeast [24], more than 30% of the 155 proteins interacting with *Pho4 *TF [26] are classified as ribosome function related according to SlimGO annotation. In *N. crassa*, 7 ribosomal proteins are regulated by *Nuc-1*, and 4 of them contain CACGTG in their promoter regions [29]. The CACGTG motif is also enriched in the upstream promoter regions of genes in cytoskeleton cluster 1,4 and cortex cluster 3. Therefore we hypothesize that in addition to Pi metabolism, *Pho4 *TF also regulates genes belonging to diverse functional categories including protein biosynthesis and the cell cycle.

#### Stress response

The other top scoring motif M3, ACGTCA, matches the binding site of *Sko1 *in *Sc *and *Atf1 *in *Sp*, both of which are known TFs that regulate response to environmental stress [30,31]. Even though both *Sko1 *and *Atf1 *are known to use the same TF binding sites (ACGTCA), their roles in stress defense and the set of genes they regulate have significantly diverged. *Sko1p *in *Sc *regulates a small set of genes in response to osmotic shock [30], while its ortholog *Atf1 *in fission yeast *Sp *regulates a larger gene set induced by environmental stress under diverse conditions that define *Atf1 *as a core environmental stress response (CESR) regulator [32]. There is a *Sko1*/*Atf1 *ortholog in each *Fusarium *genome (FGSG_10142, FVEG_02866, FOXG_05265 and JGI_69482), although the *Fusarium *genes are more similar to *Atf1 *(1e-32 between FGSG_10142 and *Atf1*, 4.5e-9 between FGSG_10142 and *Sko1*). About 20 *Sko1 *target genes were identified in *Sc *[30], 9 of which are conserved in *Fg*. The binding site, M3, is conserved in 8 of them. Similar conservation and motif enrichment result is also observed among the 314 *Sp *induced CESR gene set (Figure 5). It is likely that the Sko1/Atf1 homolog in Fg regulates both CESR as in Sp and the osmotic defense response as reported in Sc. Consistent with this hypothesis, Sp is reported to be sensitive to osmotic stress, while both Fg and Sc are resistant to osmotic stress [33], which may indicate that the osmotic regulation through the Sko1 pathway may have either evolved after the split of the Sp lineage, or lost in Sp. The smaller genome size and number of genes in the two yeast systems implies that the yeasts are streamlined derivatives of a common ascomycete ancestor.

In *Sc*, the CESR is regulated through a different pathway using the duplicated TF gene pair *MSN2/4 *which bind to a sequence that differs from the *Sko1*/*Atf1 *binding site. A single *MSN *homologous gene is present in each *Fusarium *genome (FGSG_06871, FVEG_05115, FOXG_01955, and JGI_102564). The Fg genes homologous to the Sc CESR pathway genes are also enriched, but for a slightly different motif than the MSN2/4 binding sites (Figure 5). Based on all these observations, we infer that, in response to environmental stresses, the filamentous fungus *Fg *employs the combined regulatory pathways used in both yeast systems.

Large sets of genes are also repressed under environmental stress in *Sc *and *Sp *respectively [31,32] and the same TF binding site was reported in both systems. About 60% and 33% of the repressed genes, from *Sc *and *Sp *respectively, share homologous genes in *Fg*. Two very similar motifs M19 and M21 are significantly enriched in two homologous sets accordingly (Figure 5). Clearly similar regulatory mechanisms are operating among these diverse fungal genomes, while there are more genes involved the regulation in the filamentous fungus *Fg *comparing to the rather streamlined yeast regulatory systems. The functional association study also revealed that these two motifs (M19 and M21) are enriched in the Cellular Ribosome GO category, cluster 2 (Figure 3). In rapidly growing yeast cells, about 60% of total transcription is devoted to ribosomal RNA, and 50% of RNA polymerase II transcription is devoted to ribosomal proteins (RPs) [34]. Therefore repression of the genes encoding ribosomal proteins may ensure the economic use of resources under stressed conditions.

As one of the initial efforts to study whole genome regulation in filamentous fungi, it is perhaps not surprising to see many of the motifs discovered from our study have no matches in *S. cerevisiae*, including the top-scoring motif M1 (GCGGG). However, comparing these motifs to known TF binding sites from other filamentous fungi identified the M1 motif as highly similar to the binding site of transcription factor CreA (G/CPyGGGG), a well known repressor responsible for carbon catabolite repression in *Aspergillus nidulans *[35]. By searching for other known *Aspergillus *transcription factor binding sites within the list of discovered *Fusarium *promoter motifs, we found two additional matches, including motifs M5 (GCCARG) and M26 (YGATAAG), which show high similarity to the *PacC *and the *AreA *DNA-binding sites respectively. *PacC *plays an important role in mediating the response to ambient pH, while *AreA *belongs to the GATA family of DNA-binding proteins that binds to HGATAR and mediates nitrogen metabolism repression [36].

### Discovered motifs in downstream and intronic regions

In addition to the promoter motifs, our analysis also yielded a number of motifs showing significant conservation in the downstream and intronic regions (Additional file 1, Table S1), although the overall number is smaller and the motifs are less conserved when compared to the promoter region (Additional file 1, Figure S4). The most conserved intronic motif (GTNAGT, Additional file 1, Table S1), corresponding to a splice donor motif, is both abundant (6648 instances) and well conserved with C.R. (conservation rate) equal to 37%. However, among the downstream conserved motifs we didn't find the mammalian polyadenylation signal (AATAAA). The polyadenylation hexamer, though abundant, is rarely conserved in the *Fusarium *genome. Among the downstream regions, we found a total of 1138 occurrences, only 11 of which are conserved (C.R.: 0.9% and MCS = -3.69), which is even lower than the rate for a random hexamer (C.R.: 2.7%). The known polyadenylation signals in *Sc*, TATATA and TTAAGAAC [37,38], also have a low MCS value of -1.8 (6 conserved from a total of 409 instances) and -0.3 (0 conserved from a total of 9 instances) respectively, suggesting that a different polyadenylation signal may be used in the *Fusarium *genomes.

The discovered downstream motifs and the splice signals in the introns show a strong directional bias, while the promoter motifs have similar conservation rate in both the forward and reverse strands (Additional file 1, Figure S5). This directional specificity is consistent with the function of the downstream motifs and the splice signals in post-transcriptional processes, in contrast with the promoter motifs that act at the level of DNA, as binding sites during transcription.

## Discussion

There is limited knowledge about transcriptional regulation among filamentous fungi. This study is the first attempt to systematically characterize sequence signatures that are conserved through evolution and may have the potential to function as regulatory elements among several *Fusarium *species, a group of agricultural important filamentous fungi. The study results in a total of 73 conserved elements in promoter regions. These promoter motifs are enriched in the upstream region of the genes involved in specific cellular functions, indicating their potential functional association with specific biological processes and validating the biological significance of our discovery. Interestingly, we observed the conservation among *Fusarium *species and two yeast model systems, *Sc *and *Sp *for some TFs, their binding sites and target genes regulated by these TFs for some important pathways, including phosphate metabolism and stress response. Both computational and experimental approaches were used to define modularity of regulatory network [39]. The conservation of potential regulatory elements accompanying certain conserved modules was also reported [39,40].

Constantly exposed to a wide range of environmental changes, including hostile conditions, environmental stress response is essential for the survival of fungi. In that sense, it is not surprising to observe a significant conservation among both induced and repressed gene sets in response to environmental stresses across divergent lineages. Our study suggests that the core environmental stress response (CESR) is a highly conserved physiological mechanism that protects cells and organisms from stressful changes in their environment. The conservation between *Sc *osmotic stress response pathway and the *Fg *gene sets suggests that this regulatory pathway may have evolved after the split of Schizosaccharomyces, or was lost in this Ascomycete basal lineage. The more complete list of genes that are repressed under ESR in *Fg *also suggests that both yeast systems have independently reduced portions of the pathway through evolution.

The focus of this study is on short regulatory motifs with size around 7 bp. Therefore translation-related motifs, which are typically longer, are likely missed by our method. In addition, the translation-related motifs such as internal ribosome entry site (IRES) and upstream ORFs (uORFs) typically observe different conservation characteristics than the transcription-related motifs, since what is preserved for IRES is its secondary structure and what is preserved for uORFs is their short ORF structure. Detecting these translation-related motifs likely will require a new conservation measure that can take these specific characteristics into account. It is interesting to note that a motif containing ATG (ATGACGN with the MCS >30), which could encode the start codon of uORFs, is detected in the upstream regions.

## Conclusions

We developed a computational pipeline to systematically discover regulatory motifs in *Fg *by comparing its genome to three other closely related *Fusarium *genomes. Our analysis yielded 73 candidate motifs in promoter regions, 69 in the downstream regions, and 39 in the intronic regions. Out of the 73 motifs discovered in the promoter regions, 22 showed strong enrichment in functionally related gene clusters, and 16 showed strong sequence similarity to known motifs in *Sc*, altogether corresponding to 41% of the discovered motifs, suggesting that many of the motifs are likely true functional elements. Comparison of these motifs with known yeast TF binding sites revealed a significant conservation for signals involved in phosphate metabolism and ESR pathways, providing the first look into the regulation of these biological processes in *Fg*. Such conservation across millions of years of evolution since the divergence of yeast and *Fg *indicates the functional importance of these regulatory pathways. The analysis presented here demonstrates the power of comparative genomics for discovering functional elements in the *Fusarium *genome. As more genomes and better annotation of genes become available, the list of regulatory motifs presented here can be further refined.

## Methods

### Genome alignments and annotation

Local-alignment anchors were detected using PatternHunter (1e-10) [41]. Contiguous sets of anchors with conserved order and orientation were chained together within a 10 kb distance. The multiple alignments for all the syntenic regions that cover a common segment on the reference genome *Fg *were conducted using Mlogan [42] We used the annotation of *Fg *[5,6] as a reference to define the coding sequence versus intergenic (600 bp upstream of the start codon and 600 bases downstream of the stop codon) and intronic regions in the aligned regions. For the intronic regions, we extended sequences by 2 bp both upstream and downstream in order to include the intron splice sites.

### Motif Conservation Score

The motif conservation score (MCS) for the i^th ^heptamer H_*i *_is computed using the following function:

where *K*_*i *_is the number of conserved occurrences and *N*_*i *_is the number of total occurrences of H_i _in the aligned genome of *Fg *with *Fo*, *Fv *and *Fs*. *P*_*o *_is the fraction of total conserved occurrences among the total occurrences of all heptamers. The same function is used to compute the MCS for degenerate motifs. However, the *P*_*o *_is calculated differently. Since *P*_*o*_denotes the average conservation rate of a motif **m**, we scan 5000 random heptamers in the genome and calculate its conservation as per the degeneracy profile of **m**. This conservation score is then used to compute the average conservation rate for a motif whose degeneracy profile is the same as **m **(See Additional file 1 for details).

### Degeneracy

We used consensus sequences to represent regulatory motifs. The sequences are spread over 11 alphabets, consisting of four nucleotides A, C, G, T, the six two-fold degenerate characters S = [C or G], W = [A or T], Y = [C or T], R = [A or G], M = [A or C], K = [G or T], and the four-fold degenerate character N = [A, C, G, or T]. A motif **m **occurs in the *Fg *genome when each nucleotide in the genome satisfies the corresponding degenerate character in the consensus sequence of **m**. We used a greedy approach to discover motifs with higher MCS values by adding degeneracy as described in the main text and Additional file 1.

### Clustering

We clustered the degenerate motifs as per their sequence similarity. We quantify the similarity between two motifs by the Pearson correlation of their equivalent position weight matrices. The weight matrices represent the frequencies of different nucleotides at each base. For example, if the base is W = (A or T), its corresponding column in the matrix will be represented as [0.5, 0, 0, 0.5] in the order of A, C, G and T. We computed the Pearson correlation coefficient between two positional weight matrices as described in Xie et al [18].

### Enrichment

The enrichment, or overlap, between two sets of genes is computed by assuming an underlying hyper-geometric model. Given the total number of ***N ***genes in *Fg*, with ***F ***number of genes in a functional category set, ***M ***number of genes that contains a motif **m**, and ***K ***number of genes that are shared by ***F ***and ***M***, the *P-value *of enrichment of motif **m **in the set ***F ***is computed as:

Since this value is usually very close to zero, we have used -log_10_(*P-value*) to denote the enrichment. For the later analysis of overlap between the set of genes derived as homologs from other eukaryotic species namely *Sp *and *Sc*, the ***N ***variable is limited to the number of genes in that species that have clear orthologs in *Fg*. Similarly, ***F ***and ***M ***are also reduced to only include genes with clear orthologs in *Fg*. Since only about a quarter of genes have orthologs in *Fg*, this reduction allows us to compute the effective enrichment without biasing the function towards the genes that are present in set but have no orthologs in *Fg*.

### Determining one-to-one orthologous genes

We derived orthologous genes from *Sc *and *Sp *to the genes in *Fg *for our gene set enrichment analysis based on a one-to-one map from yeast genes to the gene in *Fg *according to the highest similarity score of Blastp (E ≤ 10^-5^).

### Strains and materials

The sequenced strain of *F. graminearum *(PH-1; [5]) was used for expression analysis. Gene expression levels were determined using a custom *Fusarium graminearum *Affymetrix microarray (Fusariuma520094; [11]). Data from a spore germination time-course were used to create expression clusters [10] and raw data are available as experiment FG7 at the database PLEXdb [43]. K-means clustering was performed in the Analyst module of Expressionist version 5.1 (Genedata) using raw data imported and normalized according to [10]. The silhouette score was used to determine the optimum number of clusters for genes in each GO category using a specified range of 2-5. Default settings were used for distance, centroid calculation and maximum iterations fields.

## Authors' contributions

LK implemented and executed the computational tools for motif discovery and promoter enrichment analyses; AB performed the gene expression and clustering analysis; CK, LM and XX designed and coordinated the project; all authors wrote the paper; all authors read and approved the final manuscript.

## Supplementary Material

Additional file 1 **Supplementary methods on motif discovery**. This file contains a more detailed description of the methods used in motif discovery. Click here for file
