# Supplementary material for: Systematic discovery of regulatory motifs in Fusarium graminearum by comparing four Fusarium genomes
Source: BMC Genomics. 2010 Mar 26;11:208. doi: 10.1186/1471-2164-11-208 (PMC2853525; doi:10.1186/1471-2164-11-208)
Supplement: Additional file 1 — Supplementary methods on motif discovery. This file contains a more detailed description of the methods used in motif discovery. [file 1471-2164-11-208-S1.PDF]

Supplementary Information

S1. Motif Discovery

Heptamers We used the genomic sequence of *F. graminearum* aligned with sequences of *F. oxysporum*, *F. verticillioides* and *F. solani* species in Fusaria genus as the data input for the discovery of over-conserved heptamers. Our method computes two counts for each possible heptamer occurring in the aligned sequence; namely the (i) number of occurrences (***N***) of that heptamer in the reference genome *Fg* and (ii) the number of occurrences (***K***) that are also conserved in other species. An occurrence of a heptamer is defined as conserved if the same heptamer also at the aligned location in other Fusaria species. Since the alignment may not be perfect, we expanded the aligned location by 5bp outwards from both the ends to search for the occurrence of the identical heptamer.

Scanning the entire input dataset for ***N*** and ***K*** is computationally expensive to repeat for every heptamer.. We thus check for conservation of heptamer in the first 7bp of input sequences. This 7bp window is then shifted ahead by 1 bp and the heptamer in new window is checked for conservation. This process continues till we reach end of the sequences. The window of 7bp may increase at times to adjust for the gaps introduced in the sequences for alignment. Our method does not allow more than 5 gaps in the window, thereby limiting the size of window to 12.

We next define the probability of conservation or conservation rate (CR) of Heptamer $\mathbf{H_i}$ as:

$$CR_i = P_i = \frac{K_i}{N_i}$$

where K_i is the number of conserved occurrences of $\mathbf{H_i}$ out of its total N_i occurrences. The average probability of conservation of any random heptamer **H** is defined as:

$$CR_o = P_o = \frac{\sum_i K_i}{\sum_i N_i}$$

We thus compute the Motif conservation score (MCS) for $\mathbf{H_i}$:

$$MCS_i = \frac{K_i - N_i P_o}{\sqrt{N_i P_o (1 - P_o)}}.$$

where we divide the excess conservation of $\mathbf{H_i}$ by the standard deviation assuming binomial distribution to be the null model. The complete list of heptamers is provided as Supplementary data with the paper and is also available for download from our project website.

A histogram of scores is shown in Figure 1 and Figure S1 for upstream, downstream and intronic regions respectively. We would expect a random input dataset to produce a histogram curve similar to normal distribution. The positional bias of histograms in Figure 1 and S1 towards positive scores suggests presence of functionally significant heptamers among higher scoring ones. We next selected

heptamers with MCS value above 4.4 to represent over conserved heptamers. This threshold, 4.4, is chosen, as it's the absolute value of the lowest score in the histogram for upstream region. This threshold provides 688 over conserved heptamers in the promoter, 326 in the downstream and 234 heptamers in the intronic region. Figure S2 shows the overlap of these heptamers among the three regions.

We were also interested to know if the high scoring heptamers are common across the regions. A relative asymmetrical distribution of the MCS values of heptamers for each pair of the region, shown in Figure S4, shows that the heptamers with high MCS values are not shared among the regions. This suggests that the over-conserved heptamers region specific and potentially play significant role in their region. Figure S4 a,c also shows a denser presence of promoter heptamers among high scores as compared to heptamers in downstream and the intronic region.

Degeneracy We have used consensus sequences to represent regulatory motifs. The sequences are spread over 11 alphabets, consisting of four nucleotides A, C, G, T, the six two-fold degenerate characters S=[CG], W=[AT], Y=[CT], R=[AG], M=[AC], K=[GT], and the four-fold degenerate character N=[ACGT]. A motif **m** is said to occur in the *Fg* genome at a site when each nucleotide in the site satisfies the corresponding degenerate character in the consensus sequence of **m**.

The greedy approach to increase the MCS values by adding degeneracy is provided in the main text. An overview of how our method computes the average conservation rate of a motif **m** is described in the Methods section. Here, we show how we identify if an occurrence of a heptamer **H** in the aligned genome is conserved or not as per the degeneracy profile of the **m**. If, for example, the first character of **m** is a two-fold degenerate character, and the first nucleotide of **H** is A, we convert the nucleotide to a two-fold degenerate character that contains A; namely W, R or M. We repeat this process for every next character of **m**. This may convert **H** into more than one motif as more than one degenerate characters are possible for a two-fold degeneracy. All these resulting motifs are then checked for their conservation in the three aligned species and **H** is considered as conserved if any one of them is conserved.

We also observed that most of the discovered promoter motifs are strand symmetric while the motifs discovered in the 3'-UTR region were specific to either forward or reverse strand (Figure S5).

Clustering We clustered the degenerate motifs as per their sequence similarity. We quantify the similarity between two motifs by the Pearson correlation of their equivalent position weight matrices. The weight matrices represent the frequencies of each nucleotides at each base, for example if the base is W=(A,T), its corresponding column in the matrix would be [A C G T] = [1/2 0 0

1/2]. We next compute the correlation as described in the supplementary section of Xie et al [1]. Next we admit motifs into a cluster if they are at least 0.75 correlated (within a range of -1 to +1) with the highest scoring motif of that cluster. We do this by iterating over motifs in the decreasing order of their MCS values. We join a motif in a cluster of previously visited motifs if it satisfies the threshold with that cluster; else create a new cluster of its own. Table S1 shows the top 25 motifs discovered in the downstream and the intronic region.

S3. Promoter enrichment with gene clusters

We compute an enrichment score of each motif with respect to all the 68 functional gene clusters. The high enrichment of the motifs in multiple functional categories (Figure 2) indicates their presence as biologically significant for the gene clusters. We were also interested to validate the statistical significance of the scores by comparing them with some randomly chosen motifs. We, thus, permuted all the degenerate motifs, by mapping their nucleotides A, C, G, T to, respectively, C, T, A, G in the consensus sequence of that motif. The two-fold degenerate characters in the consensus sequence were also changed to maintain the mapping done for the nucleotides. For example, S=[CG] was changed to W=[TA] as the nucleotide C was mapped to A and G was mapped to T in the sequence. We repeat our enrichment analysis with the set of permuted motifs. We found a very less percentage of permuted motifs (12%) that had a reasonable enrichment (P-value < 10^{-3}) as compared to the discovered motifs (56%). This contrast increases further for higher enrichment (P-value < 10^{-5}), as only 2% of the permuted motifs are above this threshold when compared to 28% discovered motifs, suggesting a false discovery rate of 7% among the promoter motifs.

S4. Association with known TF binding site regulatory pathways

We used the online STAMP program [2] to identify the known Transcription factor (TF) binding sites in the *S. cerevisiae* that are similar in sequence with the motifs discovered in *Fg.* We repeated this analysis for permuted motif to understand the statistical significance of the similarity scores. For a high score threshold of E-value < 10^{-7}, we found 14 (19%) different motifs satisfying it (Table 2). In contrast, 5 permuted motifs satisfied this criterion. This indicates a false discovery rate of 35%.

Orthologous gene sets specific to biological states Certain sets of genes have been reported to respond under environment stress in *Sc* [3] and *Sp* [4]. We downloaded these sets from their website supplements [5, 6]. We next created a one-to-one mapping of all orthologous genes among *Sc* and *Fg,* and *Sp* and *Fg* (see methods). We used this mapping to derive corresponding sets of stress response genes in *Fg*.

We also downloaded a set of genes from the *Saccharomyces* Genome Database [7] that are known to interact physically or genetically with the transcription factors listed in Table 2 and similarly mapped

them to their orthologous pair in *Fg*. The enrichment results of *PHO4* (phosphate metabolism) and *SKO1* (osmotic stress response) are shown in Table 3.

References

1. Xie XH, Lu J, Kulbokas EJ, Golub TR, Mootha V, Lindblad-Toh K, Lander ES, Kellis M: **Systematic discovery of regulatory motifs in human promoters and 3 ' UTRs by comparison of several mammals.** *Nature* 2005, **434**(7031):338-345.
2. Mahony S, Benos PV: **STAMP: a web tool for exploring DNA-binding motif similarities**. *Nucleic Acids Res* 2007, **35**(Web Server issue):W253-258.
3. Gasch AP, Spellman PT, Kao CM, Carmel-Harel O, Eisen MB, Storz G, Botstein D, Brown PO: **Genomic expression programs in the response of yeast cells to environmental changes**. *Mol Biol Cell* 2000, **11**(12):4241-4257.
4. Chen D, Toone WM, Mata J, Lyne R, Burns G, Kivinen K, Brazma A, Jones N, Bahler J: **Global transcriptional responses of fission yeast to environmental stress**. *Mol Biol Cell* 2003, **14**(1):214-229.
5. **Genomic responses of Yeast to Diverse Stress Conditions** [http://genome-www.stanford.edu/yeast_stress/]
6. **Stress response data of Schizosaccharomyces Pombe** [http://www.bahlerlab.info/projects/stress/]
7. ***Saccharomyces* Genome Database** [http://www.yeastgenome.org/]

Table S1 Top 20 discovered motifs in downstream and intron

Downstream			Intron		
Motif	CR	MCS	Motif	CR	MCS
GGMGTT	0.29	71.2	GTnAGT*	0.36	172.5
ATACCC	0.33	62.0	AGGTnnG*	0.44	152.9
CCCCRC	0.24	29.4	GnGTAnG*	0.36	110.1
CACGTG	0.26	26.8	CnGTAnG*	0.31	98.4
GGCGnnG	0.13	25.0	TnGTAnG*	0.26	85.3
TGRCGT	0.13	23.2	TGGTnAG*	0.29	71.1
KAKACCA	0.15	20.8	GCTnAC¶	0.16	57.6
TGATAC	0.11	19.2	CCCCnC	0.23	45.2
CGGnCCG	0.19	18.6	GGTnCGT	0.16	37.3
AnGAnGA	0.07	18.0	GnTGAG	0.12	26.0
CCAATnR	0.11	17.1	CCCGCC	0.21	23.3
CnGGGG	0.10	16.8	TGGnTnG	0.11	21.3
CTCCnC	0.10	16.5	CGTCAT	0.16	20.9
TGCAT	0.07	15.8	GnGTGnG	0.13	20.1
GCGnTnG	0.09	15.6	GTGGnnG	0.11	14.5
CCnCTC	0.09	14.6	TGCSCC	0.12	14.3
AGCCnC	0.08	14.1	CAATTGR	0.11	14.2
CnCGGC	0.09	14.0	ATGGC	0.09	13.5
GGATMCC	0.15	13.8	GAnTGG	0.09	12.6
GCGnGnG	0.09	13.4	GnCGCG	0.11	12.4

The top 20 motifs of the total 69 discovered motifs in the downstream and 39 discovered motifs in the intron region. The absence of the mammalian polyadenylation signal AATAAA in the downstream motifs suggests that a different signal is used in Fusarium. The splice donor sites in the intron motifs show exceptionally high MCS values. *: motifs matching splicing donor sites; ¶: the motif matching the branch site.

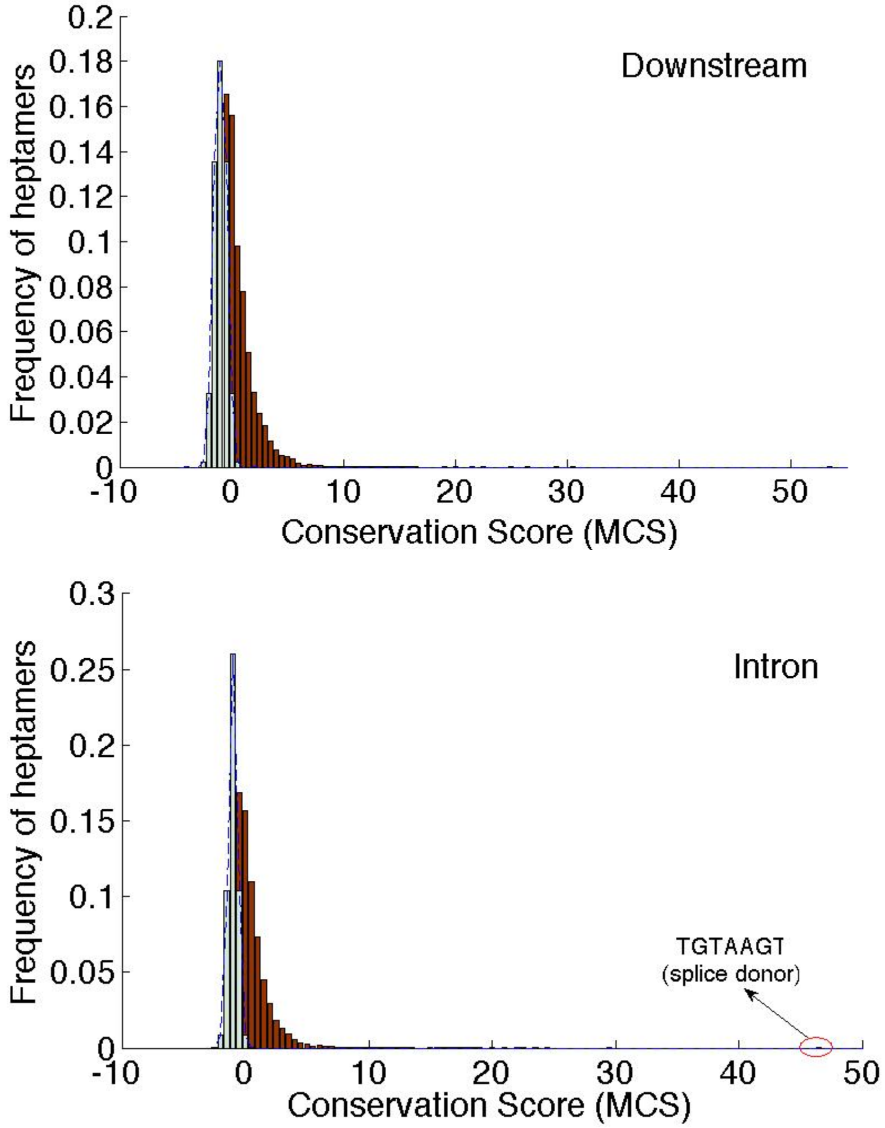

Figure S1 Conservation properties of 7-mers in *Fusarium* downstream and intronic regions. Excess conservation in the downstream (top panel) and intronic region (second panel) is shown by a histogram of the MCS values of heptamers. The dashed curve is a close approximation of a Gaussian distribution. Excess conservation is shown by the red shaded region outlying the dashed curve.

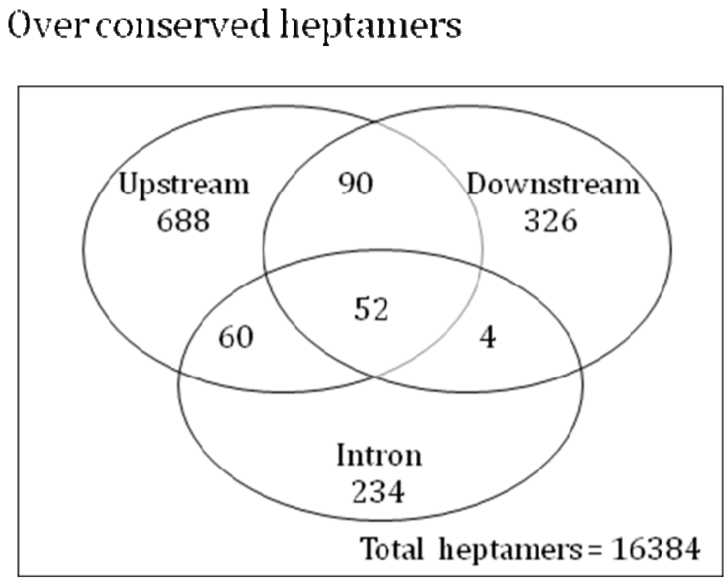

Figure S2 Venn diagram of the over-conserved heptamers.

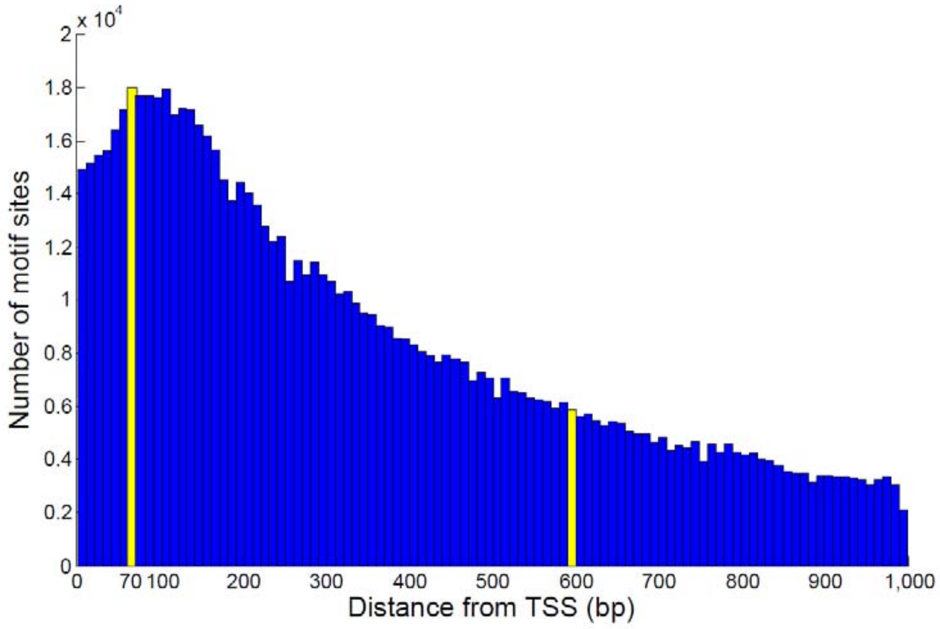

Figure S3 Locations of the discovered motif sites relative to gene starts in promoter regions

The promoter dataset used for this figure was defined as 1000bp upstream of gene starts. The histogram shows that most (> 80%) of the motif sites are located within 600bp from gene starts with the highest density located at 70bp.

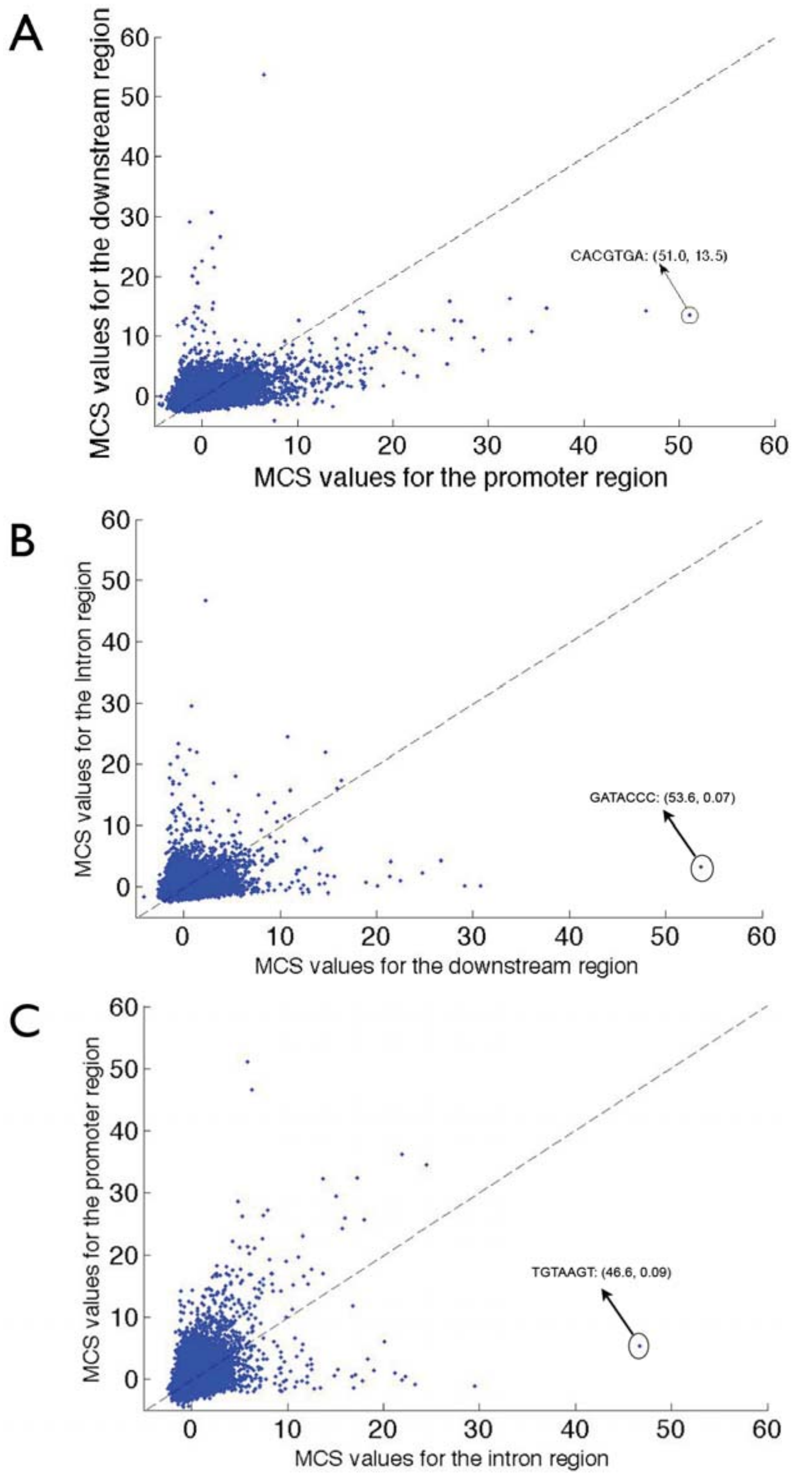

Figure S4 Comparison of the MCS values of 7-mers between different genomic regions

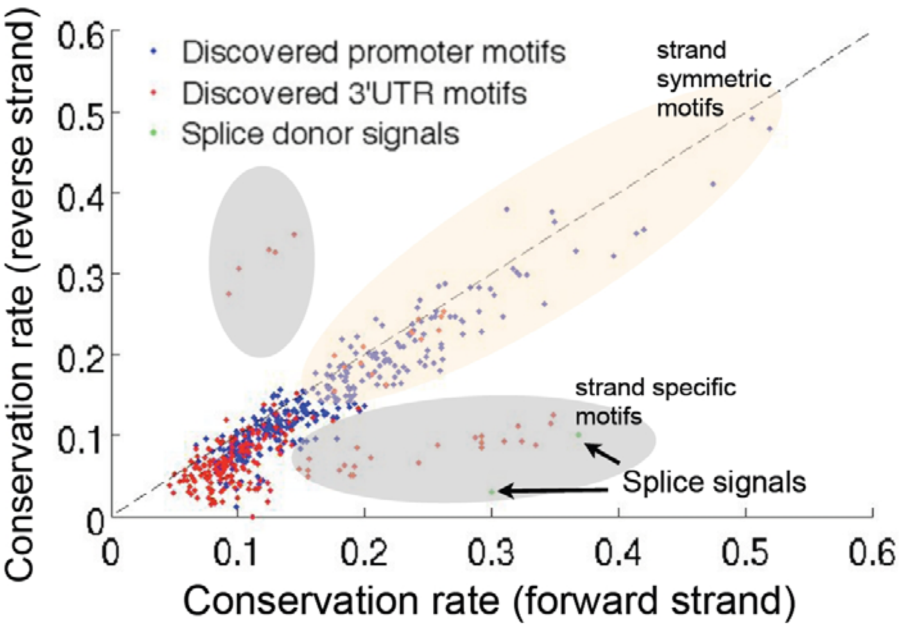

Figure S5 Strand specificity of the discovered motifs

Conservation rates of motif sites located in the forward strand (X-axis) are plotted against the conservation rates of motif sites located in the reverse strand (Y-axis). For each motif the reverse complement was excluded from the figure.
